# Comparative in-vivo toxicity of venoms from South Asian hump-nosed pit vipers (Viperidae: Crotalinae: *Hypnale*)

**DOI:** 10.1186/1756-0500-5-471

**Published:** 2012-08-29

**Authors:** Anjana Silva, Panduka Gunawardena, Danister Weilgama, Kalana Maduwage, Indika Gawarammana

**Affiliations:** 1 Department of Parasitology, Faculty of Medicine and Allied Sciences Rajarata, University of Sri Lanka, Saliyapura, Sri Lanka; 2 Department of Veterinary Pathobiology, Faculty of Veterinary Medicine and Animal Science, University of Peradeniya, Peradeniya, Sri Lanka; 3 Department of Parasitology, Faculty of Medicine and Allied Sciences Rajarata, University of Sri Lanka, Saliyapura, Sri Lanka; 4 School of Medicine and Public Health, University of Newcastle, NSW, Australia; 5 Department of Medicine, Faculty of Medicine, University of Peradeniya, Peradeniya, Sri Lanka

**Keywords:** Hypnale, Nepa, Zara, Venom, Toxicity, Histopathology

## Abstract

**Background:**

Envenoming by south Asian hump-nosed pit vipers (Genus: *Hypnale*) is a significant health issue in Sri Lanka and in peninsular India. Bites by these snakes frequently lead to local envenoming, coagulopathy and acute renal failure even resulting in death. Recently the genus was revised and the existence of three species *viz H. hypnale, H. nepa* and *H. zara* were recognized. There is, however, a paucity of information on the toxicity of the venoms of these species. Hence, we compared the toxic effects of the three *Hypnale* venoms using BALB/c mice.

**Findings:**

Intraperitoneal median lethal doses (LD_50_) for *H. hypnale*, *H. zara* and *H. nepa* venoms were 1.6, 6.0 and 9.5 μg protein/g respectively. Minimum haemorrhagic doses for venoms of *H. hypnale*, *H. zara* and *H. nepa* were 3.4, 11.0 and 16.6 μg protein/mouse respectively. The minimum necrotic doses for the same venoms were 15.0, 55.1 and 68.2 μg protein/mouse respectively. Severe congestion and petecheal haemorrhages were observed in lungs, kidneys, liver and the alimentary tract. Histopathogical examination of kidneys revealed proximal tubular cell injury and acute tubular necrosis with intact basement membrane indicating possible direct nephrotoxicity. *Hypnale* venoms caused pulmonary oedema, hepatocellular degeneration and necrosis, focal neuronal degeneration in brain and extramedullary haemopoiesis in spleen. *H. hypnale* venom caused all above histopathological alterations at lower doses compared to the other two.

**Conclusion:**

*Hypnale* venoms cause similar pathological changes with marked differences in the severity of the toxic effects in vivo. Therefore, differences in the severity of the clinical manifestations could possibly be seen among bite victims of the three *Hypnale* species.

## Findings

### Background

Hump-nosed pit vipers (genus: *Hypnale*) are a group of small sized pit vipers restricted to Sri Lanka and Western Ghats of South India. These snakes are the commonest cause of snake bite in Sri Lanka [1] and also lead to medically significant envenoming in Sri Lanka [2] and in India [3]. Systematics of the genus *Hypnale* was recently revised by Maduwage et al. [4] and three species namely, *H. hypnale*, *H. nepa* and *H.zara* were established. Each of the three species of *Hypnale* show unique geographical distribution and habitat characteristics [4].

Local envenoming is the commonest clinical effect among *H. hypnale* bite victims and could range from mild swelling to severe gangrene of bitten site [2]. Coagulopathy and renal failure have also been reported in many of *H. hypnale* victims [2,5,6] and in one authenticated *H. zara* envenoming [7]. Clinical reports on *H. nepa* and *H. zara* bites, however, are extremely rare. Concerns on frequent potentially fatal envenoming caused by *H. hypnale* bites has been raised recently [2]. Although lethality, haemorrhagic and necrotic activity and several enzymatic activities of *H. hypnale* venom has been reported [8,9], such information is not available on the other two venoms. Recently, Maduwage et al. [10] showed the reverse phase high performance liquid chromatography profiles of the three *Hypnale* venoms to be similar. Further, they demonstrated similar potent cytotoxic, weak procoagulant, neurotoxic, myotoxic and phospholipase A_2_ activities in all three *Hypnale* venoms. However, no clinical or basic in vivo experimental studies are available on the venom toxicity of *H. nepa* and *H. zara*.

The present study compares the Lethality (LD_50_), haemorrhagic and necrotic activity of the three *Hypnale* venoms and describes *Hypnale* venom induced pathological changes in major organs of BALB/c mice.

### Materials and methods

#### Venom collection, preparation and storage

The specimens of *Hypnale* species identified using keys described in Maduwage et al. [4] were used for venom collection.Venom was collected from the following: *H. hypnale*, 6 specimens from Galle (06°03’N, 80°12’ E, elevation: 15 m) and 2 specimens from Peradeniya(07°15’N, 80°36’ E, elevation: 465 m), *H. nepa* (4 specimens) from Agrapathana (06°50’N, 80°40’ E, elevation: 1665 m) and *H. zara* (4 specimens) from Kottawa (06°06’N, 80°20’ E, elevation: 25 m). The crude venom was desiccated using dehydrated silica gel in polyethylene containers. Venom crystals were dissolved in 0.9% sterile NaCl solution and 1% stock venom solutions prepared. For experiments, venoms were used within 6 months of the collection.

#### Protein assay

Since the pooled samples of the three Hypnale venoms were directly crystallized, quantification of venom proteins was performed in order to standardize the three venoms

Standardiztion was done using Bradford protein assay method [11]. BSA standard 0 to 2.0 mg/ml (Bio-Rad, USA) was prepared from dilution of a 2 mg/ml stock solution. Undiluted 300 μl of Bradford Reagent (Sigma, Germany) was mixed with 10 μl of venom sample in a microplate and allowed to develop for 5 minutes at room temperature before absorbance measurements at 595 nm in microplate reader (Molecular Devices, USA). A blank was prepared by using 10 μl of sterile 0.9% NaCl solution. All standards and samples were duplicated.

All doses of venom mentioned in this paper refer to respective venom protein doses expressed either as μg/g or μg/mouse.

#### Mice

BLAB/c mice of both sexes, 10–12 weeks old, weighing 18–23 g reared at Animal facility, Faculty of Medicine and Allied Sciences, Rajarata University, Sri Lanka were used. The animals were handled according to the guidelines given by CIOMS on animal experimentation [12]. Ethical clearance for the study was obtained from the Ethics Review Committee of the Faculty of Medicine and Allied Sciences, Rajarata University.

#### Assessment of lethality

In lethality studies, venoms were injected intraperitoneally with varying venom protein doses ranging from 0.1to 11.5 μg/g of mouse in a total volume of 300 μl. Following initial dose adjustment studies, median lethal doses (LD_50_) of the three *Hypnale* venoms were ascertained by using six venom doses with five mice per each dose of each venom. Number of mice that died within 48 hours of envenoming in each test group was recorded. A control group of eight mice were injected with a similar volume of the diluent. The LD_50_ values with respective 95%confidence intervals (CI) were calculated using probit analysis method as described by Finney [13] using SPSS software version 17.0. The minimum venom dose that killed all the animals of a test group was considered as the minimum lethal dose (MLD).

#### Assessment of haemorrhagic activity

Haemorrhagic activity of the venoms was estimated using the method followed by Tan et al. [8].Groups of mice (for each venom: 5 groups, 3 per group) were injected intra-dermally with three *Hypnale* venoms in varying amounts diluted in sterile 0.9% NaCl solution. Each mouse received a standard venom volume of 40 μl in to the shaved dorsal skins. Mice were kept under light diethyl ether anesthesia with access to water and food until they were sacrificed at 90 minutes of venom injection. The dorsal skins of mice were removed and the minimum and maximum diameter of the haemorrhagic lesion on the inner side of the skin was immediately measured to the nearest 0.1 mm using a dial venire caliper (KWB, Switzerland). The mean of the diameter readings of the haemorrhagic lesions were plotted against the corresponding venom protein dose injected to each group and trend-lines for each *Hypnale* venom was drawn. Venom protein dose corresponding to a diameter of 10 mm was considered as the minimum haemorrhagic dose (MHD) [8].

#### Assessment of necrotic activity

Necrotic activity of venoms was estimated using the method followed by Tan et al. [8]. Mice (3 per group) were injected with varying doses of *Hypnale* venoms as described for haemorrhagic activity tests and were sacrificed after 72 hours. The mean diameter of the necrotic lesion on the inner side of skin was measured as described for haemorrhagic activity tests. The mean of the diameter readings of the necrotic lesions were plotted against the corresponding venom dose and trend-lines for each *Hypnale* venom was drawn. Venom protein doses corresponding to a necrotic lesion with a diameter of 5 mm was considered as the minimum necrotic dose (MND).

#### Gross and histopathological studies

Necropsies were performed on a total of 60 mice envenomed with venoms of the three *Hypnale* species (20 per each venom) during lethality studies. The selected mice were representative of full dose range tested in the lethality studies and had different survival times. In addition, three mice that survived envenoming with each of the *Hypnale* venom were euthanized after 7 days necropsied. Control mice groups (2 mice per group) were euthanized at 2 hours, 12 hours, 24 hours and 7 days following injection of 300 μl doses of sterile 0.9% NaCl solution intra-peritoneally and necropsies performed. At necropsy, gross appearance of liver, kidneys, stomach, intestines, heart, lungs, brain and spleens were observed and whole specimens were fixed in 10% formol saline at least for 1 week. They were sectioned and histologically processed, embedded in paraffin, and 4–5 μm sections were made in rotary microtome. Sections were stained with Haematoxylin and Eosin. Stained sections were examined using light microscopy separately by PG and AS, without knowing the type and doses of venoms used. Micrographs were obtained using a digital photo-micrographic unit, (CU318, Micrometrics, USA).

### Results

#### Lethality, haemorrhagic and necrotic activity of Hypnale venoms

Median lethal doses (LD_50_) with 95% confidence intervals, MLD, MHD and MND of the three *Hypnale* venoms are given in Table 1. All three *Hypnale* venoms showed varying degrees of haemorrhagic and necrotic activities. *H. hypnale* venom had lowest LD_50_, MHD and MND values of all three venoms, followed by *H. zara* and then *H. nepa*.

**Table 1:** **Lethality, hemorrhagic and necrotic activities of the three *Hypnale *venoms**

	***H. Hypnale***	***H. nepa***	***H. zara***
**LD**_**50 **_**(CI) (μg/g)**	1.6 (1.5–1.8)	9.5 (8.6–10.3)	6.0 (4.3–7.0)
**MLD (μg/g)**	2.0	11.4	8.7
**MHD (μg/mouse)**	3.4	16.6	11.0
**MND (μg/mouse)**	15.1	68.2	55.1

#### Hypnale venom induced pathological changes in mouse organs

**Figure 1:**
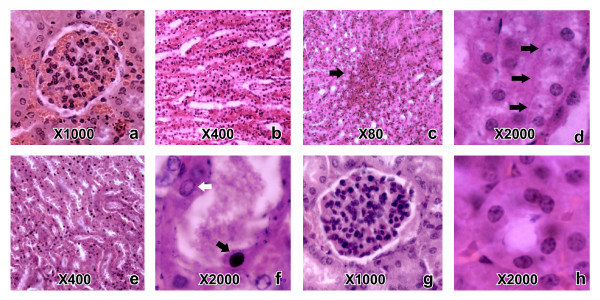
**Histopathological changes caused by *H. hypnale, H. nepa and H. zara *venoms in mouse kidneys. ** (Note: the histopathological changes caused by the three *Hypnale* venoms were similar. The dose and the type of venom led to each histopathological change in representative photographs are mentioned within the parenthesis) Congested glomeruli (**a:** 2.3 μg/g dose of *H. hypnale* venom) and peritubular vasculature (**b:** 2.3 μg/g dose of *H. hypnale* venom); petechial haemorrhages in renal paranchyma(**c:** 2.5 μg/g dose of *H. hypnale* venom); hydrophic degeneration of tubular cells (**d:** 7.0 μg/g dose of *H. zara* venom); tubular necrosis (**e:** 9.0 μg/g dose of *H. zara* venom)(note dilated tubules, many tubular cells with high cytoplasmic eosiophilia and pyknotic nuclei); dilated and necrosed proximal tubule (**f:** 9.0 μg/g dose of *H. zara* venom) filled with hyaline material and having tubular cells with pyknosed nuclei (black arrow) and a vesicular nuclei (white arrow) as seen in test mice. Normal glomerulus (**g:** 0.9% sterile NaCl solution) and proximal tubule (**h:** 0.9% sterile NaCl solution) seen in control mice.

**Figure 2:**
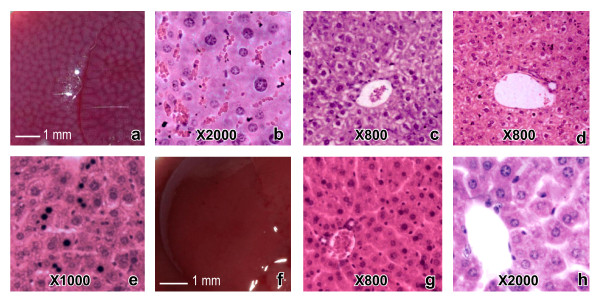
**Gross and histopathological changes caused by *H. hypnale, H. nepa and H. zara *venoms in mouse livers. ** (Note: the pathological changes caused by the three *Hypnale* venoms were similar. The dose and the type of venom led to each pathological change in representative photographs are mentioned within the parenthesis) Gross (**a:** 2.5 μg/g dose of *H. hypnale* venom) and histopathological (**b:** 2.5 μg/g dose of *H. hypnale* venom) appearances of a highly congested liver (note the highly congested hepatic sinusoids). Vacuolar degeneration of hepatocytes in centrilobular (**c:** 7.0 μg/g dose of *H. nepa* venom) and peri-portal (**d:** 4.2 μg/g dose of *H. zara* venom) patterns. Random hepatocellular necrosis (**e:** 2.8 μg/g dose of *H. zara* venom) as evident by presence of pyknoticneuclei and eosinophiliccytoplasms in some hepatocytes. Gross (**f:** 0.9% sterile NaCl solution) and histological (**h:** 0.9% sterile NaCl solution) appearances of a control mouse liver.

**Figure 3:**
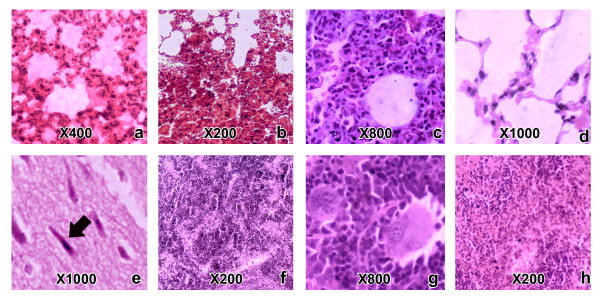
**Histopathological changes caused by *H. hypnale, H. nepa and H. zara *venoms in mouse lungs, brains and spleens. ** (Note: the histopathological changes caused by the three *Hypnale* venoms were similar. The dose and the type of venom led to each histopathological change in representative photographs are mentioned within the parenthesis) Note severe pulmonary oedema (a: 2.3 μg/g dose of *H. hypnale* venom), pulmonary interstitial haemorrhage (b: 3.0 μg/g dose of *H. hypnale* venom), inflammatory cell infiltrate in alveolar septae (c: 1.4 μg/g dose of *H. hypnale* venom) in lungs of test mice. Normal alveoli of a control mouse are shown in figure (d: 0.9% sterile NaCl solution). Ishchemic neuronal degeneration in cerebral cortex is shown in figure (e: 5.3 μg/g dose of *H. zara* venom). Hyperplasia of the red pulp of spleen (f: 1.5 μg/g dose of *H. hypnale* venom) as evident by large aggregations of immature stages of erythrocytes and presence of megakaryocytes (g: 6.0 μg/g dose of *H. nepa* venom) seen in splenic red pulp of test mice. Red pulp of a control mouse is shown in (h: 0.9% sterile NaCl solution).

Gross and histopathological changes were observed in all organs examined except the heart. Changes observed in kidney, liver, lung, brain, spleen and intestine were similar with all three *Hypnale* venoms.The minimum dose of each *Hypnale* venom that led to each histopathological change differed drastically (Table 2). No macroscopic or microscopic pathological alterations were noted in any of the above organs of the control mice (Figures 1g, 1h, 2f, 2g, 2h and 3d).

**Table 2:** **Minimum doses of the three *Hypnale *venoms led to each observed histopathological alteration in BALB/c mouse organs**

**Histopathological change**	**Minimum venom dose (μg/g)**		
	***H. hypnale***	***H. nepa***	***H. zara***
Petecheal haemorrhages in renal paranchyma	0.9	4.8	3.2
Degenerative changes in renal tubules	0.4	2.9	2.2
Renal tubular necrosis	1.2	8.0	4.4
Degeneration of hepatocytes	0.5	3.8	2.1
Random hepatocellular necrosis	0.7	5.2	2.8
Congestion in lung paranchyma	0.3	3.6	2.2
Haemorrhage into alveolar spaces	1.3	5.1	3.7
Inflammatory infiltrate in alveolar sepate	1.3	4.8	3.7
Pulmonary oedema	1.6	7.6	3.0
Focal neuronal degeneration in brain	1.8	8.2	5.3

#### Kidney

Macroscopically, congestion and petecheal haemorrhages in medulla were observed.

Microscopically, congestion of glomeruli (Figure 1a) and peritubular vasculature (Figure 1b),petecheal haemorrhages in renal medulla (Figure 1c),degenerative changes in tubular cells (Figure 1d), tubular dilatation and flattening of tubular cells with interrupted tubular brush border were observed in mice that died as early as 3 hours of envenoming. Renal tubular necrosis (Figure 1e) was evident by nuclear pyknosis and shedding of the tubular cells into lumen (Figure 1f) predominantly in the proximal tubular cells. Necrosed tubules had intact basement membranes and these were evenly distributed within cortices. Tubular necrosis was extensive in mice that died 18 to 72 hours of envenoming. Glomerular changes were mild and were mainly restricted to congestion, dilatation and presence of hyaline material in Bowman’s capsule.

#### Liver

In macroscopy, oedema and congestion were commonly seen. Severe congestion with mottled appearance(Figure 2a) was seen in some mice. Microscopically, congestion of liver sinusoids (Figure 2b) and centrilobular vacuolar degeneration of hepatocytes (Figure 2c) was consistent at low doses. Peri-portal vacuolar degeneration of hepatocytes (Figure 2d) was present, less commonly. Few necrosed hepatocytes with random distribution (Figure 2e) were present in some sections. No haemorrhages were observed macroscopically or microscopically even at the highest venom doses tested.

#### Lung

Macroscopically, pulmonary congestion, gross haemorrhages and petecheal haemorrhages were evident. Pulmonary congestion, oedema (Figure 3a) and petecheal haemorrhages (Figure 3b) were observed microscopically. The latter was observed, even at venom doses less than LD_50_ values. Inflammatory cell infiltrate predominately with lymphocytes and with few polymorphoneuclear cells in alveolar septae and peribronchial areas was observed (Figure 3c).

#### Brain

Focal areas of neuronal degeneration (Figure 3e) were seen in cerebral gray matter of mice envenomed with all *Hypnale* venoms.

#### Spleen

Spleens were highly congested, oedematous and friable in mice that died. However, in those that survived for 7 days, gross spleenomegaly was evident, at times being about double the normal size. In microscopy, petecheal haemorrhages were seen in red pulp of spleen in few of the mice following envenoming by *H. hypnale* venom. Presence of numerous small islands of darkly stained cells in red pulp of subcapsular area (Figure 3f), presence of numerous aggregations of megakaryocytes (Figure 3g) and large aggregates of haemosiderin engulfed macrophages were seen in spleens of mice, after 7 days post envenoming.

#### Stomach, small and large intestine

Severe congestion was noted macroscopically and microscopically, in stomach and in the small and large intestines of mice envenomed with all three *Hypnale* venoms.

### Discussion

The present study suggests that the *in-vivo* toxicity of the *Hypnale* venoms to be different among the three species. In comparison with other two venoms, markedly low LD_50_ value of *H. hypnale* venom suggests that the overall venom toxicity of this species is higher*.* Further, *H. hypnale* venom was found to be more haemorrhagic and necrotic, than the other two species. All three venoms however, had similar pathological effects on kidney, liver, lung, brain, spleen, stomach and intestines. Renal tubular necrosis with insignificant glomerular changes, degeneration of hepatocytes, pulmonary oedema and haemorrhage, patchy neuronal degeneration in cerebral grey matter and evidence of extramedullary haemopoiesis in spleen were the major histopathological findings in mice. The minimum venom doses that resulted in pathological alterations of mouse organs suggest that *H. hypnale* venom is more toxic to these organs than the other two venoms. This indicates a possible difference in the severity of envenoming among victims bitten by different *Hypnale* species.

Evenly distributed renal tubules with degeneration and necrosis, presence of intact basement membranes in severely necrosed tubules and absence of disarrayed tubular architecture suggest direct nephrotoxicity due to *Hypnale* venoms. Similar observations have been reported by Gunatilake et al. [14], using rabbit kidney slice model. With the results of this study, it could be suggested that venoms of all three *Hypnale* species are capable of causing direct tubular injury. Maduwage et al. [4] demonstrated the potent *in-vitro* cytotoxic effects of three *Hypnale* venoms. Various cytotoxic components in snake venom lead to direct tubular injury in kidney [15]. Thus, such properties may have contributed to direct tubular injury in envenomed mice.

Bilateral renal cortical necrosis has been observed in *Hypnale* victims previously [4,16]. In addition, evidence for presence of thrombotic microangiopathy in seven of eleven *Hypnale* bite victims with severe acute kidney injury was described recently [17]. Renal histology of these patients showed multiple glomerular capillary thrombi along with necrosis of glomeruli and tubular epithelial cells. Recently, the role of thrombotic microangiopathy in causing acute renal injury in most snake bites, predominately in viperid bites was emphasized [18]. However, none of the mouse kidneys examined in this study had evidence of the effects of thrombotic microangiopathy. In any case, the etiology and mechanisms of developing thrombotic microangiopathy in snake bite victims has yet to be unraveled [18].

Severe local necrosis is a well documented feature of *H. hypnale* bite victims [2,19]. However, since venoms of *H. nepa* and *H. zara* too showed necrotic activity in this study, local necrosis is likely to be associated with the bites caused by these two species. Potent cytotoxicity and Phospholipase A_2_ activity has been detected in all three *Hypnale* venoms [4], hence such prediction could be further justified.

Compared to the present study, intraperitoneal LD_50,_ MHD and MND values for *H. Hypnale* reported by Tan et al. [8,9] were different and it is difficult to comment on this as the latter authors have not commented on the protein quantification.

Presence of haemorrhages in lungs and kidneys with doses below the respective LD_50_ values and absence of such in livers even with the highest respective venom doses tested might indicate organ specific haemorrhagic activity of *Hypnale* venoms. Pulmonary haemorrhage has not been observed in patients bitten by hump-nosed pit vipers as yet. It is known that metalloproteinases found in the snake venom are capable of inducing the release of inflammation mediators such as cytokines, intensifying the inflammatory response [20]. The occurrence of a large quantity of inflammatory cells in alveolar septae of mice may be due to such activity of the three venoms.

Degeneration and necrosis of hepatocytes observed in mice livers indicate hepatotoxicity in all three *Hypnale* venoms. Evidence for such activity has been observed in victims of *H. hypnale* bites [16,21] and a victim of *H. zara* bite [7]. Barraviera et al. [22] hypothesized that hepatic injury as a frequent result of Crotaline envenomings.

Peri-portal area is the first area of the hepatic lobule to be exposed to a toxin being delivered through the bloodstream via the portal vein [23]. Compounds administered via intraperitoneal route are absorbed primarily through the portal circulation and, therefore, passes through the liver before reaching other organs [24]. Therefore, it could be surmised that peri-portal hepatocellular degeneration observed in mice are likely to be due to the effects of the venom absorbed via portal system.

Areas of neuronal degeneration in cerebral cortices of mice resembled ischemic neuronal degeneration. However, neither evidence of haemorrhage nor of thrombosis was revealed in these sections. Hence it is difficult to comment on the cause of these changes.

Presence of numerous small islands of darkly stained erythropoietic precursor cells seen in grossly enlarged spleens of mice, 7 days following envenoming, indicate extramedullary erythropoesis in spleen. Presence of numerous aggregations of megakaryocytes in red pulp also indicates production of platelets in spleen. Thrombocytopenia and anaemia have been observed in *Hypnale* victims previously [17]. The above observation indicates a response by spleen to such haemtological changes by hyperplasia and extramedullary haemopoesis.

Although the toxicity of the three *Hypnale* venoms were found to be clearly different as revealed in this study, these differences may not be much obvious in clinical situations. This is because the amount of venom injected during actual snake bite is subjected to large variations [25]. Several factors including size, sex, age of the snake also affect the toxicity of venom as well as the injecting venom volume in snake bite [25]. As clinical reports on *H. nepa* and *H. zara* envenomings are scarce [7] for the comparison, prospective clinical studies with accurately authenticated *Hypnale* bites would be helpful in correlating these findings to actual clinical situations.

### Conclusions

There is a lacuna of information on species specific envenoming by hump-nosed pit vipers. *In-vivo* toxicity studies conducted on mice has shown that there is an appreciable variation in the toxicity of the venoms amongst the species of genus *Hypnale*. There is a need for detailed characterization of *Hypnale* venoms and the components. Future clinical reporting should appreciate species specific clinical effects of *Hypnale* envenoming.

## Abbreviations

LD_50_: Medial lethal dose; MHD: Minimum haemorrhagic dose; MLD: Minimum lethal dose; MND: Minimum necrotic dose.

## Competing interests

The authors declare that they have no competing interests.

## Authors’ contributions

AS, DW, PG and IG designed the study, AS, DW and KM conducted experiments, AS and PG conducted histopathological studies, all authors participated in writing of the manuscript. All authors read and approved the final manuscript.
